# Progressive arteriopathy with vasospasm in focal cerebral arteriopathy in childhood: a case report

**DOI:** 10.1186/s12883-023-03334-z

**Published:** 2023-07-24

**Authors:** Takuma Nishimoto, Fumiaki Oka, Natsumi Fujii, Hirofumi Inoue, Shunji Hasegawa, Masatoshi Yamane, Sadahiro Nomura, Hideyuki Ishihara

**Affiliations:** 1 Department of Neurosurgery, Yamaguchi University School of Medicine, 1-1-1, Minamikogushi, Ube, Yamaguchi, Yamaguchi, 755-8505 Japan; 2 Department of Pediatrics, Yamaguchi University Graduate School of Medicine, Yamaguchi, Japan; 3 Department of Radiology, Yamaguchi University Hospital, Yamaguchi, Japan

**Keywords:** Focal cerebral arteriopathy, Progressive arteriopathy, Vasospasm, 3D-SPACE

## Abstract

**Background:**

Focal cerebral arteriopathy (FCA) is a clinically important disease that often causes progressive arteriopathy. We report a case of FCA with progressive arteriopathy due to arterial shrinkage of the outer diameter found on T2-weighted three-dimensional sampling perfection with application optimized contrasts using different flip angle evolutions (3D-SPACE) imaging.

**Case presentation:**

The patient was a 9-year-old girl who developed right hemiparesis. Acute infarction was detected in the basal ganglia. Vascular images revealed stenosis from the distal internal carotid artery (ICA) to the middle cerebral artery (MCA). Intravenous heparin was administered for 8 days, and the symptoms improved. However, 29 days after onset, right hemiparesis transiently developed again and magnetic resonance angiography (MRA) showed progressive stenosis from the ICA to MCA, while 3D-SPACE showed similar shrinkage of the outer diameter. Aspirin was started, and there was no subsequent recurrence. After 12 months, MRA and 3D-SPACE showed improvement of stenosis and arterial shrinkage.

**Conclusions:**

Given the time course, the change in the outer diameter was thought to be vasospasm. Thus, vasospasm may be one of the causes of progressive arteriopathy in FCA.

## Background

Focal cerebral arteriopathy (FCA) of childhood (also known as transient cerebral arteriopathy (TCA)) is a relatively common cause of acute ischemic stroke [1]. FCA is diagnosed radiologically using vascular imaging and is defined as unifocal and unilateral stenosis or irregularity of the large intracranial arteries: the internal carotid artery (ICA) and/or its proximal branches [2]. FCA is subdivided into FCA-inflammation (FCA-i), FCA-dissection and FCA-undetermined types. FCA-i is diagnosed when the case is thought to be due to inflammation (i.e., focal vasculitis) or when evolution of the arteriopathy is typical of TCA: a stereotypical, monophasic natural history characterized by frequent early progression (from days to weeks), a plateau with nonprogression by 6 months, and subsequent improvement in some cases and complete resolution in a minority of cases [2].

FCA is a clinically important condition that often progresses to arteriopathy, which increases the risk of recurrent stroke [3]. Such progressive arteriopathy can be evaluated based on internal caliber using computed tomography angiography, magnetic resonance angiography (MRA) and digital subtraction angiography (DSA). Progressive arteriopathy has been considered to be associated with vessel wall thickening evaluated by vessel wall enhancement (VWE) in vessel wall imaging [3]. However, the correlation of progressive arteriopathy with the vascular outer diameter has not been widely reported.

Heavily T2-weighted magnetic resonance imaging (MRI) is useful for displaying the margin between the cerebrospinal fluid (CSF) and intracranial structures, including the details of the arterial wall [4]. A recent study showed the change in the outer diameter in the involved arteries in moyamoya disease using heavily T2-weighted MRI [5]. T2-weighted three-dimensional sampling perfection with application optimized contrasts using different flip angle evolutions (3D-SPACE) imaging is one of the heavily T2-weighted MRI techniques that uses a 3D fast spin echo that permits high-resolution imaging of complex anatomy. 3D-SPACE shows the course of blood vessels as distinct low-signal areas within regions of high-signal CSF and can visualize an occluded artery (as a low signal) that cannot generally be identified on MRA [6, 7].

Herein, we report a case of FCA with the development of progressive arteriopathy with arterial shrinkage of the outer diameter (a so-called vasospasm) found on 3D-SPACE.

## Case presentation

**Fig. 1:**
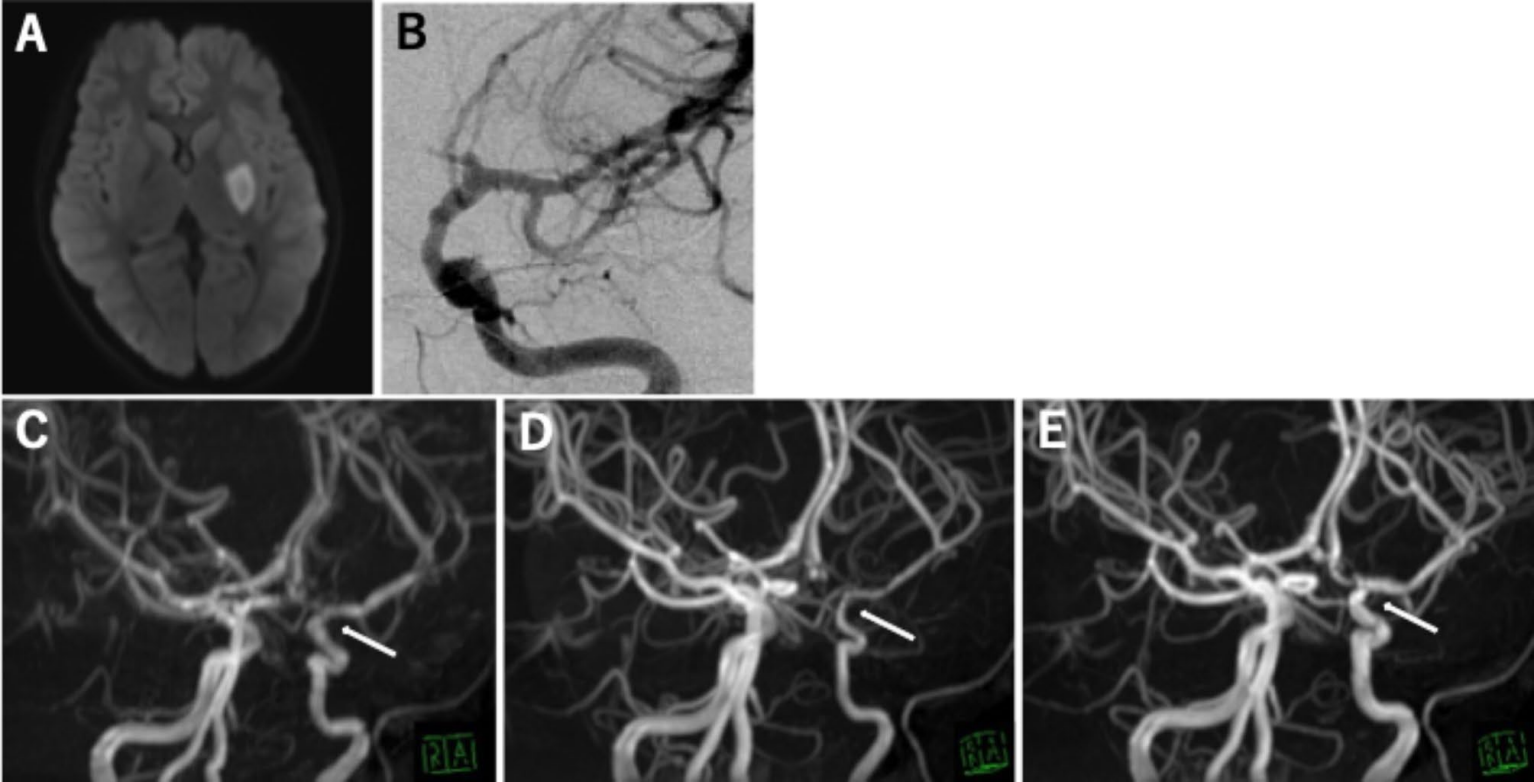
A case of focal cerebral arteriopathy-inflammation in a 9-year-old girl. (**A**) Axial diffusion-weighted image on admission, showing an acute infarct involving the basal ganglia in the lenticulostriate artery territory. (**B**) Digital subtraction angiography of the left internal carotid artery (ICA) 7 days after admission, showing irregular stenosis with a banding pattern from the distal ICA to the middle cerebral artery (MCA) and occlusion of the anterior cerebral artery (ACA). (**C**) Magnetic resonance angiography (MRA) on admission, showing slight stenosis from the ICA to the MCA and ACA (arrow). (**D**) MRA at 29 days after onset, showing worsened stenosis from the distal ICA to MCA and occluded ACA (arrow). (**E**) MRA at 12 months after onset, showing improvement of the internal caliber from the ICA to the MCA and ACA (arrow)

**Fig. 2:**
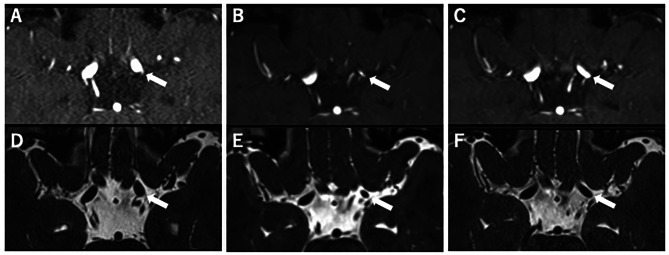
Relationship between internal caliber and outer diameter in the arteriopathy at the left internal carotid artery (ICA) C1-2 segment (arrow). (**A**) Magnetic resonance angiography (MRA) on admission, showing slight luminal stenosis. (**B**) MRA at 29 days after onset, showing progression of luminal stenosis. (**C**) MRA at 12 months after onset, showing improvement of luminal stenosis. (**D**) 3D-SPACE on admission, showing slight arterial shrinkage at the stenotic lesion. (**E**) 3D-SPACE at 29 days after onset, showing worsened arterial shrinkage at the stenotic lesion. (**F**) 3D-SPACE at 12 months after onset, showing improvement of the arterial shrinkage of the outer diameter. The changes in the outer diameter on 3D-SPACE were almost the same as those in the internal caliber on MRA.

A 9-year-old girl developed right hemiparesis and was admitted to our department at approximately 8 h after onset. She did not have medical history, operation history, and prescribed medication. And she also did neither complain headache or transient ischemic symptom. On arrival, vital signs were normal. Initial diffusion-weighted (DWI) magnetic resonance imaging showed acute infarction in the basal ganglia in the lenticulostriate artery territory (Fig. 1A). MRA showed mild stenosis from the distal ICA to the middle cerebral artery (MCA) and anterior cerebral artery (ACA) (Figs. 1C and 2 A), and 3D-SPACE showed mild shrinkage of the outer diameter with the same internal caliber as that found on MRA (Fig. 2D). At 7 days after admission, DSA of the left ICA indicated mild irregular stenosis with a banding pattern from the distal ICA to MCA and occlusion of the ACA (Fig. 1B).

After admission, examinations were performed to investigate the cause of cerebral arteriopathy. Serum levels of sodium, potassium, chloride, albumin, and the lipid profile were within normal limits. The complete cell count was also normal. The homocysteine level in blood was also within normal limit. Autoimmune tests including antinuclear antibody, antineutrophil cytoplasmic antibody, anti-single and anti-double-stranded DNA IgG antibody, rheumatoid factor, and anticardiolipin antibody were all negative or within normal limits. High-sensitivity C-reactive protein was not measured, but the C-reactive protein level was normal. Serum virus antibodies, including those for herpes simplex virus, varicella zoster virus, Epstein-Barr virus, cytomegalovirus, and human immunodeficiency virus, were also within normal limits. The cardiac evaluations including 12-lead electrocardiography, echocardiography, 24-hrs Holter monitoring electroencephalography showed no abnormal findings. Moreover, cervical MRA or DSA showed no evidence such as Takayasu’s arteritis or carotid dissection. After admission, intravenous injection of unfractionated heparin (100 IU/kg/day) was started for 8 days. Although mild stenosis remained, right hemiparesis gradually improved, and the patient was discharged without neurological deficits after 15 days. However, 29 days after onset, right hemiparesis transiently developed again. DWI showed no acute infarction, but MRA showed progressive stenosis from the distal ICA to the MCA in left cerebral hemisphere (Figs. 1D and 2B). Moreover, 3D-SPACE showed worsening of arterial shrinkage of the outer diameter in parallel with the change in luminal stenosis on MRA (Fig. 2E). In this study, 3D-SPACE was performed with a 3.0-Tesla (Magnetom Skyra; Siemens AG, Germany) instruments and was acquired with the following parameters: repetition time, 1,500; echo time, 203 msec; flip angle, 120º; matrix size, 384 × 384; slice interval, 0.6 mm; field of view, 180 mm; voxel size, 0.5 × 0.5 × 0.6 mm; Average, 2; and acquisition time, 4 min 53 s.

Aspirin was started at 100 mg/day, and there was no subsequent recurrence of ischemic symptoms or progressive arteriopathy for 12 months. At 12 months after onset, MRA showed improvement of luminal stenosis from the ICA to MCA and ACA in left cerebral hemisphere (Figs. 1E and 2 C), and 3D-SPACE also showed improvement of corresponding shrinkage of the outer diameter (Fig. 2F). Because the course of arteriopathy met the diagnostic criteria for TCA, she was diagnosed with FCA-i, which includes TCA [2].

## Discussion and conclusions

We report a case of FCA-i with progressive arteriopathy that showed specific changes in the outer diameter that were similar to those in the internal caliber. Thus, progressive arteriopathy in FCA-i may be associated with arterial shrinkage of the main lesion of the large intracranial arteries of the anterior circulation. Given the time course, the change in the outer diameter was thought to be vasospasm. This is the first report to suggest a relationship between progressive arteriopathy in FCA-i and a change in the vascular outer diameter on 3D-SPACE.

The exact mechanisms of vasospasm during progressive arteriopathy in FCA-i are unclear, but the characteristics of FCA-i, such as an inflammatory response and a main lesion of the large intracranial arteries of the anterior circulation, may be associated with vasospasm. The etiology of FCA-i mainly involves an aberrant inflammatory response. In a histopathologic study, CNS inflammatory disease was found to cause concentric inflammatory endothelial disruption [8]. This dysfunction decreases the levels of vasodilators and leads to vasospasm in several vascular diseases [9]. Therefore, an inflammatory response in FCA-i may cause concentric inflammatory endothelial disruption and lead to vasospasm.

FCA lesions mainly occur in the large intracranial arteries of the ICA and/or its proximal branches. Anatomically, these arteries are surrounded by CSF, and in a healthy child the arteries do not have vasa vasorum [10]. In contrast, in pathological conditions such as vascular diseases with an inflammatory response, the vasa vasorum develops at these arteries [11], and a recent study has shown that the vasa vasorum is associated with vasospasm [12]. Therefore, an inflammatory response in the FCA-i may cause the vasa vasorum to develop in arteries surrounded by CSF, which may lead to vasospasm.

Progressive arteriopathy in the FCA has also been linked to vessel wall thickening evaluated by VWE [3], and concentric inflammatory endothelial disruption and development of the vasa vasorum are also associated with VWE [11, 13]. Thus, in FCA-i, endothelial dysfunction or development of the vasa vasorum may lead to progressive arteriopathy due to both vessel wall thickening and vasospasm.

In the case of FCA-i reported here, we found a relationship between luminal stenosis and vasospasm in progressive arteriopathy. Vasospasm may be one of the causes of progressive arteriopathy in FCA-i, and investigation of the vascular outer diameter may be useful for understanding the pathological status of the disease. Further studies in larger numbers of patients are required to understand the pathophysiology of progressive arteriopathy in FCA.

## Data Availability

All data and material supporting the conclusions of this article are included in the article. Identifying/confidential information has not been and shall not be shared.

## Abbreviations

FCA: focal cerebral arteriopathy
TCA: transient cerebral arteriopathy
ICA: internal carotid artery
FCA-i: FCA-inflammation
MRA: magnetic resonance angiography
DSA: digital subtraction angiography
VWE: vessel wall enhancement
MRI: magnetic resonance imaging
CSF: cerebrospinal fluid
3D-SPACE: three-dimensional sampling perfection with application optimized contrasts using different flip angle evolutions
DWI: diffusion-weighted magnetic resonance imaging
MCA: middle cerebral artery
ACA: anterior cerebral artery
